# Health care professional’s communication through an interpreter where language barriers exist in neonatal care: a national study

**DOI:** 10.1186/s12913-019-4428-z

**Published:** 2019-08-19

**Authors:** Katarina Patriksson, Helena Wigert, Marie Berg, Stefan Nilsson

**Affiliations:** 1 Institute of Health and Care Sciences, Sahlgrenska Academy, University of Gothenburg, Arvid Wallgrens Backe, Box 457, S-405 30 Gothenburg, Sweden; 2 Division of Paediatrics, NÄL Hospital, S-461 85 Trollhättan, Sweden; 3 Division of Neonatology, Sahlgrenska University Hospital, S-416 85 Gothenburg, Sweden; 4 Norra Älvsborgs Länssjukhus, Lärketorpsvägen, S-46185 Trollhättan, Sweden

**Keywords:** Healthcare professional, Interpreter, Neonatal, Quantitative, Survey

## Abstract

**Background:**

A number of parents in neonatal care are foreign-born and do not speak the local language, which makes communication between healthcare professionals and parents more difficult. Interpreters can be used when language barriers exist - parent interactions, medical communication and communication about the care of the child*.* The aim in this study was to examine healthcare professionals’ use of interpreters and awareness of local guidelines for interpreted communication in neonatal care.

**Method:**

A survey was distributed to all 2109 employees at all 38 neonatal units in Sweden, thus to all physicians, registered nurses and nurse assistants in active service. Data were analysed with descriptive statistics and dichotomized so the professionals were compared in groups of two using the Mantel-Haenszel Chi Square test and Fisher’s Non Parametric Permutation test.

**Results:**

The survey was answered by 41% (*n* = 858) representing all neonatal units. The study showed a difference between the professional groups in awareness of guidelines, availability of interpreters, and individual resources to communicate through an interpreter. Nurse assistants significantly lesser than registered nurses (*p* < .0001) were aware of guidelines concerning the use of interpreters. In emergency communications nurse assistants used authorized interpreters to a significantly lesser extent than physicians (*p* < .0001) and registered nurses (*p* < .0001). Physicians used authorized interpreters to a significantly higher extent than registered nurses (*p* 0.006) and non-authorized interpreters to a significantly lesser extent than registered nurses (*p* 0.013). In planned communications, nurse assistants used authorized interpreters to a significantly lesser extent than physicians (*p* < .0001) and registered nurses (*p* < .0001). Nurse assistants rated their ability to communicate with parents through an interpreter to a significantly lesser extent than physicians (*p* 0.0058) and registered nurses (*p* 0.0026). No other significant differences were found.

**Conclusion:**

The results of the study show insufficient awareness of guidelines in all neonatal units in Sweden. Clinical implications might be to provide healthcare professionals with guidelines and training clinical skills in using interpreters and increasing the availability of interpreters by having interpreters employed by the hospital.

**Electronic supplementary material:**

The online version of this article (10.1186/s12913-019-4428-z) contains supplementary material, which is available to authorized users.

## Background

In Sweden, all people have access to health care regardless of geographical area and socioeconomic or educational level. Health care is free of charge for all newborn infants. When an infant requires neonatal care it is common for the infant’s parents to stay at the hospital [1]. Daily communication occurs between health care professionals and the parents of a newborn infant who is being treated in the neonatal care unit [2]. In neonatal care, parents are supported in learning about their baby’s behaviour and how to respond to the baby’s cues through supported involvement and participation in care-giving activities [3]. In the case of parents who are foreign-born and do not speak Swedish, the success of the communication between health care professional and non-Swedish-speaking parents depends on the health care professionals being aware of and having access to the guidelines on communication using an interpreter and, further, on health care professional tapping into their own personal resources, such as their own language skills [4]. With global migration an increasing number of people are residing in countries where another language is spoken. Of these, 70% are between 20 and 65 years of age, which means that a large number of immigrants are of childbearing age [5]. In 2017 in Sweden, out of a 10 million population, 1.6 million were foreign-born and 25,600 of these were asylum seekers [6]. According to the Swedish Health and Medical Service Act the healthcare professional can use interpreters when needed without economic aspects [1], interpreters should be used to ensure the patients’ rights. Interpreters are used in consultations between health care professionals and foreign-born parents about the infant’s care and treatment in the neonatal care unit. There is no current understanding of the extent to which physicians, registered nurses and nurse assistants in neonatal care are aware of the guidelines applicable to interpreted communications, and the extent to which interpreters are used is unknown [4].

According to the Swedish Health and Medical Service Act [1], the healthcare professionals have different roles. Nurse assistants are responsible for the basic care, registered nurses are responsible for the advance care and physicians are responsible for the medical care. Health care professionals are responsible for ensuring that parents are afforded the opportunity to be present during and involved in their child’s care. When health care is provided to children, the best interests of the child must be taken into account and the child’s guardians, in most cases the parents, must be kept informed about their child’s care and treatment.

According to the Swedish health care law, all health care should be individualized regardless of the individual’s ethnicity and cultural background [1]. Verbal communication affects people’s understanding of their situation [7], and in health care this often involves giving and receiving information. Studies show that non-verbal communication must be combined with verbal communication, where necessary using community interpreters in communication to avoid any misunderstanding [8], as there is a risk, due to language barriers, that important information may be missed in meetings with parents of foreign origin [9]. Earlier studies also show that health care professionals may have difficulty talking through an interpreter because they lack practical experience [10], which may explain why some health care professionals prefer not to use interpreters or care for patient groups with language barriers [11]. In one study investigating patient preferences, face-to-face interpretation was the preferred method of interpretation in health care because this way the patients were able to see the interpreter’s verbal as well as non-verbal language. In uncomplicated care situations, telephone interpretation was the desirable method. Telephone interpretation is more anonymous for the participants and sometimes easier to access in emergencies [12].

Another study has shown that it takes nearly twice as long to talk via an interpreter and time is often limited in health care [12]. Training in interpreted communication can help registered nurses work more effectively through interpreting, while guidelines can support them in communication with families [11]. In recent years, there has been an increase in the use of state-certified interpreters. This has been ascribed to the increase in use of telephone interpreters [13]. There are benefits to be gained from good planning before starting an interpreted communication, including the ability to select an interpreter based on the situation and to use an interpreter with training in medical interpreting [14]. An overview concerning access in health care to support for foreign languages (i.e. non-Swedish communication) showed that there is a shortage of interpreters and especially of qualified, trained interpreters. As well, there is no clear definition of who is allowed to call themselves an interpreter and a lack of coordination between county councils and regions concerning interpreting issues [15]. In this study, “authorized interpreter” refers to an employed interpreter hired through an interpreter service. A guideline is a regulatory for when, where and how, in this case, conversations via an interpreter should be offered to the parents when language barriers are present.

### Aim

The aim of this study was to examine health care professionals’ use of interpreters and awareness of local guidelines for interpreted communication in neonatal care.

## Method

### Participants and setting

The study was a cross-sectional study including a written survey to examine awareness of guidelines and use of interpreters by health care professionals at the national level (Additional file 1). All 38 neonatal units in Sweden were divided into levels I-III, where level I was basic neonatal care, level II was specialist neonatal care and level III was neonatal intensive care [16]. The professionals at all neonatal units included physicians, registered nurses and nurse assistants. The number of surveys distributed was equal to the number of employees in each neonatal unit: a total of 2109 professionally active physicians, registered nurses, and nurse assistants. The health care professionals in the neonatal units used telephone and face-to-face interpreting.

### Data collection

#### Development of survey

The contents of the survey are purely developed for this study and were validated through a focus group led by the first author (K.P.), which included the professional categories participating in the survey study. For the focus group, one physician, one registered nurse and one nurse assistant were selected from one neonatal unit in west Sweden. All questions in the survey were read through and discussed using the think-aloud method [17]. The discussion lasted 48 min and was transcribed to text. The survey was revised based on the results from the focus group discussion to prevent subsequent misinterpretation of the questions. The survey consisted of several parts including: a section containing sociodemographic items, as well as sections asking about communication, emergency care/planned care, interpreting (face-to-face/telephonic), guidelines for interpreted communication at their unit, and the respondents’ self-assessed own ability to speak through an interpreter. The questions were answered on a Likert scale (always/often/not so often/never) or a dichotomized (yes/no) scale. The participants were also given the opportunity to motivate their answers in their own words.

#### Distribution of the survey

A paper survey regarding the use of interpreters was sent to the department heads of all neonatal departments in Sweden registered in the National Quality Registry for Neonatal Care by mail [18]. On receipt of the paper survey, the head of each unit then distributed the survey to all employees. Data collection took place during the period of March to October 2016. After about a month, respondents were reminded by telephone and after further 2 weeks’ personal visits were made to four neonatal departments to ensure that responses were obtained from all neonatal departments in Sweden.

### Data analysis

Demographic data and descriptive statistics were analysed in SPSS Statistics for Windows, version 24 [19]. In the analysis of the survey questions, interpreted communication that concerned the infants’ medical and nursing care needs was reported by physicians, registered nurses and nurse assistants in their responses to the survey. The answers were first dichotomized, and the dichotomized answers were then compared between the health care professionals. For comparison between groups, the Mantel-Haenszel chi-square test and Fisher’s non-parametric permutation test were used for ordered categorical variables using SAS version 9.3 [20]. The significance level was *p* < 0.05.

### Ethical considerations

The head of each department gave their oral and written consent, and they informed the health care professionals’ group that participation in the study was voluntary and that the aim was to examine health care professionals’ attitudes to communicating with parents in neonatal care in Sweden. Respondents gave their consent by completing the survey, choosing which questions to answer and every survey was coded to ensure that the same person could not answer the survey twice. The respondents were informed that the results of the study would be analysed at the group level; that no individual would be able to be identified; and that data would be used for research purposes only [21]. No ethical approval was sought for this study. The study complies with Swedish law concerning ethical review of research involving humans [22] and the World Medical Association and Declaration of Helsinki’s principles for medical research involving human subjects, whose purpose is to protect individuals and ensure respect for human dignity [23].

## Results

### Sample characteristics

The survey was distributed to physicians, registered nurses and nurse assistants in neonatal care and was answered by 858/2109 (41%) (Tables 1 and 2).

**Table 1:** Characteristics of healthcare professionals by level

				Test between groups *p*-value		
Variable	Level I	Level II	Level III	Level I vs Level II	Level I vs Level III	Level II vs Level III
Respondents, *n* = 858	204	486	168			
Percentage based on total response frequency	23.8	56.6	19.6			
Age, m (range) SD	42.5 (18.8; 66.7) 11.8	42.1 (20.3; 65.9) 12.0	41.7 (21.3; 64.9) 11.8	0.66	0.50	0.72
Missing	7	9	1			
Distribution men/level, m (%)	11 (5.5%)	14 (2.9%)	16 (9.5%)	0.16	0.20	0.0018*
Missing	4	4	0			
Spoke a third language/level, m (%)	36 (17.8%)	88 (18.2%)	42 (25.0%)	0.84	0.11	0.088
Missing	2	2	0			
Years in profession, m (SD)	15.2 (12.4)	15.4 (12.0)	14.9 (12.3)	0.86	0.83	0.68
Missing	17	32	12			

*p < 0.05

**Table 2:** Characteristics of healthcare professionals

				Test between groups*p*-value		
	Physicians	Nurses	Nurse assistants	Nurse assistants vs Nurses	Nurse assistants vs Physicians	Nurses vs Physicians
Respondents, *n* = 858	54	484	320			
Percentage based on total response frequency	6.3	56.4	37.3			
Age, m (range) SD	41.7 (26.1; 66.7) 9.5	40.6 (21.3; 65.9) 11.3	44.4 (18.8; 65.8) 12.9	< 0.0001*	0.13	0.51
Missing	1	6	10			
Distribution men/professional, m (%)	21 (39.6)	14 (2.9)	6 (1.9)	0.51	< 0.0001*	< 0.0001*
Missing	1	4	3			
Spoke a third language/professional category, m (%)	21 (40.4)	87 (18)	58 (18.1)	0.33	< 0.0001*	< 0.0001*
Missing	2	2	0			
Years in profession, m (SD)	11.9 (9.9)	13.1 (10.5)	19.4 (13.9)	< 0.0001*	0.0003*	0.46
Missing	2	19	40			
Numbers of emergency conversations with interpreters the latest month, m (SD)	5.21 (6.06)	2.24 (3.90)	1.82 (4.83)	0.22	0.0001*	< 0.0001*
Missing	2	63	62			
Numbers of planned conversations with interpreters the latest month, m (SD)	4.76 (4.46)	1.95 (3.05)	1.06 (4.84)	0.0026*	< 0.0001*	< 0.0001*
Missing	3	61	90			

*p < 0.05

The proportion of each professional category represents the distribution of professional categories working in the neonatal departments in Sweden at the time the data were collected.

Distribution of answers to the survey on the use of interpreters by physicians, registered nurses and nurse assistants in neonatal departments in Sweden.

#### Sociodemographic information

Pairwise comparisons between level I and level III, and level II and level III showed a significance difference in sex between levels II and III. No other significant differences were found (Table 1).

Pairwise comparisons between nurse assistants and registered nurses showed a significant difference in age with nurse assistants being older by 3.8 years. A significant difference was also found in sex between nurse assistants and physicians (*p* < 0.0001), and between registered nurses and physicians (*p* < 0.0001). Additionally, a significant difference was found between nurse assistants and physicians (*p* < 0.0001), and registered nurses and physicians (*p* < 0.0001), in ability to speak a third language. Differences were also seen in years in the profession between nurse assistants and registered nurses (*p* < 0.0001) and between nurse assistants and physicians (*p* 0.0003). No other differences were found (Table 2).

#### Awareness of guidelines

Many health care professionals reported little or no awareness of the existence of guidelines for the use of interpreters in communications at the neonatal unit. There was a significant difference between the professional groups as regards their awareness of guidelines (*p* 0.0001) (Table 3). Pairwise comparisons showed that, compared with the registered nurses, nurse assistants had significantly lower awareness of guidelines concerning the use of interpreters (*p* < 0.0001). No other significant differences were found.

**Table 3:** Characteristics of the survey

		Total *n* (%)	Physicians	Registered Nurses	Nurse assistants	*P* value
Are there guidelines for interpreted conversations in your department?	Yes	169 (20.5)	7 (13.2)	88 (18.5)	74 (25.0)	0.0001*
	No	257 (31.2)	12 (22.6)	185 (38.9)	60 (20.3)	
	Don’t know	399 (48.4)	34 (64.2)	203 (42.6)	162 (54.7)	
	Missing	33	1	8	24	
How often are authorized interpreters used for emergency conversations?	Always/often	421 (60.6)	44 (84.6)	271 (65.5)	106 (46.3)	< 0.0001*
	Not very often/never	274 (39.4)	8 (15.4)	143 (34.5)	123 (53.7)	
	Missing	163	2	70	91	
How often are authorized interpreters used for planned conversations?	Always/often	525 (84.1)	45 (93.8)	363 (91.4)	117 (65.4)	< 0.0001*
	Not very often/never	99 (15.9)	3 (6.3)	34 (8.6)	62 (34.6)	
	Missing	234	6	87	141	
How often are non-authorized interpreters used for emergency conversations?	Always/often	258 (36.4)	11 (20.8)	164 (38.8)	83 (35.6)	0.38
	Not very often/never	451 (63.6)	42 (79.2)	259 (61.2)	150 (64.4)	
	Missing	149	1	61	87	
How often are non-authorized interpreters used for planned conversations?	Always/often	101 (16.4)	3 (6.5)	55 (14.3)	43 (23.0)	0.0013*
	Not very often/never	516 (83.6)	43 (93.5)	329 (85.7)	144 (77.0)	
	Missing	241	8	100	133	
Healthcare professionals’ rating of their ability to communicate with non-Swedish-speaking parents through an interpreter.	Extremely weak/weak	19 (2.6)	1 (1.9)	8 (1.8)	10 (4.2)	0.0003*
	Neither strong nor weak	234 (31.7)	11 (20.8)	132 (29.5)	91 (38,2)	
	Strong/Extremely strong	485 (65.7)	41 (77.4)	307 (68.7)	137 (57.6)	
	Missing	120	1	37	82	

*p < 0.05

#### Availability of authorized interpreters

All health care professionals used authorized interpreters largely for emergency communications. There was a significant difference between the professional groups as regards their access to authorized interpreters (*p* < 0.0001) (Table 3).

Pairwise comparisons showed that in emergency situations, nurse assistants used authorized interpreters to a significantly lesser extent than did physicians (*p* < 0.0001) and registered nurses (*p* < 0.0001). Physicians used authorized interpreters to a significantly higher extent compared with registered nurses (*p* 0.006). No other differences were found.

In planned communications, nurse assistants used authorized interpreters to a significantly lesser extent than did physicians (*p* < 0.0001) and registered nurses (*p* < 0.0001). No other significant differences were found.

#### Availability of unauthorized interpreters

There was not a significant difference between the professional groups as regards their use of unauthorized interpreters in emergency situations. Regarding communications in an emergency, it appeared that physicians rarely used unauthorized interpreters to talk to parents. However, there was a significant difference between the professional groups in use of unauthorized interpreters for planned communications (Table 3) (*p* 0.0013). The health care professionals in the neonatal units used telephone and face-to-face interpreting.

Pairwise comparisons showed that in emergency situations, physicians used unauthorized interpreters to a significantly lesser extent compared with registered nurses (*p* 0.013). No other significant differences were found.

In planned communication, nurse assistants used unauthorized interpreters to a significantly higher extent than did physicians (*p* 0.013) and registered nurses (*p* 0.015). No other significant differences were found.

#### Individual resources for communicating through an interpreter

Respondents rated their ability to communicate with parents through an interpreter, revealing a significant difference between the professional groups (*p* 0.0003) (Table 3).

Pairwise comparisons showed that nurse assistants rated their ability to communicate with parents through an interpreter significantly lower compared with physicians (*p* 0.0058) and registered nurses (*p* 0.0026). No other significant differences were found.

## Discussion

The results showed that there was a difference between professional categories concerning both awareness of interpreter-related guidelines and use of authorized and unauthorized interpreters. It was more common to use authorized interpreters for describing a medical treatment, while information about nursing care was more often communicated via unauthorized interpreters. A study regarding healthcare professionals pointed out that interpreters were used almost exclusively in discussions with a physician regarding the child’s medical care, but very rarely in discussions with a registered nurse concerning nursing the child [24]. An exploration of this difference may be, as suggested by previous studies, that it is almost impossible to engage an interpreter for all nursing tasks, and it is often easier to ask a family member to interpret [4]. This can be an ethical dilemma, as there is no guarantee when a family member interprets that the health care professionals are safeguarding the patient’s rights. For example, there is often low transparency and the health care professional does not know what the family member is saying and whether their interpretation is a true interpretation [25].

Awareness of the guidelines pertaining to interpretation differed between the professional categories and was highest among registered nurses. Written guidelines for interpreted communication, if implemented and known by health care professionals, can be facilitative when language barriers arise [26]. Providing written guidelines is necessary because the need to use authorized interpreters in communication between health care professionals and parents cannot always be predicted, as shown by previous studies in paediatric care [9, 26, 27]. In Sweden, there are no guidelines on a national level, each neonatal unit develop independently their own guidelines.

Regarding the availability of interpreters, in this investigation we can report that health care professionals in all neonatal departments in Sweden used authorized interpreters, although there was a discrepancy between physicians’ and registered nurses’ communications. Studies show that when authorized interpreters are used, patients and professionals feel more secure because the interpreter has a high level of linguistic skill, understands medical terminology and has a duty of confidentiality through their training [12]. The importance of planning ahead of interpreted communications has been demonstrated in earlier studies, as has that of the choice of location where the conversation takes place. It is important not only to the family, but also from a cost-efficiency perspective [12, 28]. Interpreting has become a large item in hospital budgets and it is therefore important that these communications are informative, alleviate parents’ concerns and give them the opportunity to express their opinions and the family’s wishes for the stay during the care episode. The aim is to reduce the risk of misunderstanding that can arise when professionals and families of foreign origin do not understand each other [29].

Authorized interpreters are used for communications that concern the child’s medical needs to a greater extent than for communications that concern the child’s nursing needs. The results showed that nurse assistants used relatives/friends as interpreters to a greater extent, instead of authorized interpreters. Other studies report that a member of the family often interprets communications between patients and health care professionals [30]. When health care professionals are bilingual, they are often used as interpreters in nursing contexts [26, 31]. That health care professionals have not been trained in dealing with medical interpreting is an obstacle in these interpreted communications, which can lead to erroneous interpretations and misunderstandings in the communication [9]. An ethical dilemma also arises in the form of an imbalance of power and positioning, when one parent has better Swedish language skills than the other. When the parent with Swedish language skills interprets for the other parent, an ethical dilemma arises because the health care professional cannot determine whether the parent has misunderstood the information because of inadequate knowledge of medical terminology [29].

Sweden is becoming increasingly multicultural [31], implying many potential language barriers in medical contexts. In one study, the question was asked whether relatives should act as interpreters [12]. The results suggested that this may be preferable in simple situations. The authors found that relatives were easy to get hold of, provided a sense of security and confidence, were aware of the person’s worries and were able to provide support of a different nature than that provided by authorized interpreters. However, situations in which a relative acted as the interpreter could also result in poorer interpretation and confidentiality concerns. As well, relatives often do not understand the terminology and using children as interpreters is not recommended because of their inadequate language skills [12, 28]. Moreover, using children as interpreters may violate the inherent rights of the child [32].

In our study population it was more common to use authorized interpreters for communications about medical care, while information about nursing care was more often communicated via unauthorized interpreters. There may be alternatives to using unauthorized interpreters in communication with families in neonatal care. These alternatives may provide support in nursing care, but cannot replace an interpreter in communications concerning sensitive topics. Health care professionals in Swedish neonatal departments currently use technical aids such as Google™ translate [33].

Regarding individual resources to communicate through an interpreter, in the present study all health care professionals rated their ability to communicate with parents through an interpreter as strong. Several studies have shown that health care professionals tend to overestimate their ability and that self-assessments may be positively coloured [34]. Hence, mothers in perinatal care felt a loss of control over their situation when hindrance to communicate or understand necessary information [35]. Studies also show that there is a need and a desire for registered nurses to receive training in communication through an interpreter, and that this would reduce the risk of misunderstandings [9]. Establishing guidelines for communications via an interpreter would be one way to enable parents to be involved in their child’s care and treatment [3].

There are methodological shortcomings in this study that must be addressed. One is the non-response rate, as only 41% of health care professionals chose to answer the survey. The question about academic degrees was shown to be unclear, and this question was excluded in this study. However, the strengths of the study include that respondents from all neonatal departments in Sweden participated and that the overall number of completed surveys was relatively high. Another methodological shortcoming is that the self-reported awareness and use of interpreters by health care professionals in the survey may differ from actual use in clinical practice. Consequently, the study needs to be followed up with observations of the use of interpreters.

## Conclusion

The present results suggest that it is more common to use authorized interpreters for medical communications, while communications about nursing care are more often communicated via unauthorized interpreters. The results also show insufficient awareness of guidelines for interpreted communication in all neonatal units in Sweden. Clinical implications might include providing health care professionals with guidelines, training clinical skills in using interpreters and increasing the availability of interpreters by employing interpreters at the hospital. Further research might involve developing innovative solutions that facilitate communication in all types of health care.

## Additional file

Additional file 1: Talking with parents who don’t speak Swedish. National survey. (DOCX 104 kb)

## Data Availability

The datasets used and/or analysed during the current study available from the corresponding author on reasonable request.
