# Supplementary material for: Health care professional’s communication through an interpreter where language barriers exist in neonatal care: a national study
Source: BMC Health Serv Res. 2019 Aug 19;19:586. doi: 10.1186/s12913-019-4428-z (PMC6701045; doi:10.1186/s12913-019-4428-z)
Supplement: Supplementary file 1 — Talking with parents who don’t speak Swedish. National survey. (DOCX 104 kb) [file 12913_2019_4428_MOESM1_ESM.docx]

| 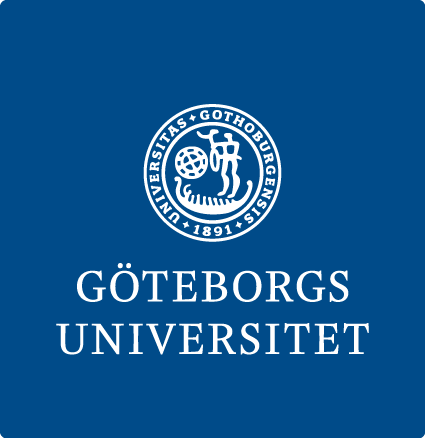 | **SAHLGRENSKA ACADEMY**  **INSTITUTE OF HEALTH AND CARE SCIENCES**  **Talking with parents who don’t speak Swedish** |
| --- | --- |

Today’s date (Year/month/day): _______________________________

Date of birth (Year/month): __________________________________________

Male □

Female □

Other □

Gender __________________________________________

Current profession/occupation __________________________________________

Physician □

With specialist training?

Yes □

No □

If yes, which specialism(s)? ____________________

Registered nurse □

With specialist training?

Yes □

No □

If yes, which specialism(s)? ____________________

Assistant nurse □

Children’s nurse □

Other occupation □

If other, which? ____________________

□

Kandidat

Bachelor □

Bachelor (advanced) □

Masters □

Doctorate □

None of the above □

Highest academic qualification __________________________________________

Your workplace __________________________________________

Your current workplace:_____________________________________________

Hospital:____________________________________________________________

Place:______________________________________________________________

Number of years at current neonatal unit:__________________________________

Number of years at other neonatal unit:____________________________________

Number of years in current profession/occupation:___________________________

Children

Do you have children, or are you the carer for one or more children?

Yes □

No □

If yes, how many children:______________

If yes, is it:

Biological □

Adopted □

Foster care placement □

Other □

_____________________________________________

Undersköterska □

Barnsköterska □

Annat yrke □

I så fall vilket annat yrke? _______________________

Do you have a partner?

Yes □

No □

If yes, how many children:______________

If yes, does your partner have children?

Yes □

No □

Language skills __________________________________________

Which language(s) can you use in conversation?

Swedish □

English □

Other □

If other, which language(s):_____________________

Conversations __________________________________________

How many emergency conversations did you have during last month, with parents who don’t speak Swedish, about the child’s medical condition/care?

Number_____________________

How often do you make use of an authorised interpreter for such conversations?

| Always | Often | Not very often | Never |
| --- | --- | --- | --- |
|  |  |  |  |

State the reasons why you always/often/not very often/never make use of an authorised interpreter.

_________________________________________________________________________________

How often do you make use of a non-authorised interpreter (other family member, member of staff, parent on the ward) during such conversations?

| Always | Often | Not very often | Never |
| --- | --- | --- | --- |
|  |  |  |  |

State the reasons why you always/often/not very often/never make use of a non-authorised interpreter.

_________________________________________________________________________________

If you use a non-authorised interpreter for these conversations, who is most commonly acting as the interpreter on these occasions?

Rank these persons, with the most common one ranked as number 1.

Another family member □

Another member of staff □

Another parent on the ward □

Another person □

State which other person _________________________

How many planned conversations did you have during last month, with parents who don’t speak Swedish, about the child’s medical condition/care?

Number_____________________

How often do you make use of an authorised interpreter for such conversations?

| Always | Often | Not very often | Never |
| --- | --- | --- | --- |
|  |  |  |  |

State the reasons why you always/often/not very often/never make use of an authorised interpreter.

_________________________________________________________________________________

How often do you make use of a non-authorised interpreter (other family member, member of staff, parent on the ward) during such conversations?

| Always | Often | Not very often | Never |
| --- | --- | --- | --- |
|  |  |  |  |

State the reasons why you always/often/not very often/never make use of a non-authorised interpreter.

______________________________________________________________________________

If you use a non-authorised interpreter for these conversations, who is most commonly acting as the interpreter on these occasions?

Rank these persons, with the most common one ranked as number 1.

Another family member □

Another member of staff □

Another parent on the ward □

Another person □

State which other person _________________________

Interpreter __________________________________________

When you make use of an authorised interpreter, what is the most common format?

Interpreter present □

Telephone interpretation □

When you make use of an authorised interpreter, is this an interpreter who is **present**?

Yes □

No □

**If yes**, how often?

| Always | Often | Not very often |
| --- | --- | --- |
|  |  |  |

State the reason why you always/often/not very often make use of an interpreter who is present.

_________________________________________________________

_________________________________________________________

**If no**, state the reason for not making use of an interpreter who is present.

_________________________________________________________

_________________________________________________________

When you make use of an authorised interpreter, is this by **telephone interpretation**? Yes □

No □

**If yes**, how often?

| Always | Often | Not very often |
| --- | --- | --- |
|  |  |  |

State the reason why your always/often/not very often make use of telephone interpretation.

_________________________________________________________

_________________________________________________________

**If no**, state the reason for not making use of telephone interpretation.

_________________________________________________________

_________________________________________________________

Guidelines __________________________________________

Does the ward provide written guidelines for interpreted conversations concerning when to make use of an interpreter?

Yes □

No □

Don’t know □

Does the ward provide written guidelines for interpreted conversations concerning the need to take account of parents’ request for a male/female interpreter?

Yes □

No □

Don’t know □

Does the ward provide written guidelines for interpreted conversations concerning where interpreted conversations should take place?

Yes □

No □

Don’t know □

Does the ward provide written guidelines for interpreted conversations concerning parents’ individual requests?

Yes □

No □

Don’t know □

Assess your ability to carry out a conversation via an authorised interpreter with parents who do not speak Swedish. Use a scale of 1–5, where 1= very poor and 5= very good.

1= very poor □

2= poor □

3= neither good nor poor □

4= good □

5= very good □

Provide reasons for your assessment.

_________________________________________________________

_________________________________________________________

_________________________________________________________
